# Solutions

**Published:** 1896-03

**Authors:** 


### Solutions.

The calculation of percentage solutions is always based upon the number of grains of water in a fluid ounce. The exact weight is four hundred and fifty-five grains, and the simplest way is to multiply this number by the percentage desired. In other words, we take one grain of the drug for every hundred grains of water. Thus to obtain a four percent solution, we multiply four hundred and fifty-five grains by four percent, which gives eighteen and two-tenths grains, or, roughly speaking, eighteen grains to the fluid ounce of water.—*Medical Brief.*

